# Prevalence and Socio-Demographic Correlates of Poor Mental Health Among Older Adults in Agricultural Areas of China

**DOI:** 10.3389/fpsyt.2020.549148

**Published:** 2020-11-05

**Authors:** Yu Jin, Yun-Shu Zhang, Qinge Zhang, Wen-Wang Rao, Li-Li Zhang, Li-Jun Cui, Jian-Feng Li, Lin Li, Gabor S. Ungvari, Todd Jackson, Ke-Qing Li, Yu-Tao Xiang

**Affiliations:** ^1^College of Education for the Future, Beijing Normal University at Zhuhai, Zhuhai, China; ^2^Department of Sleep Medicine, Hebei Psychiatric Hospital, Baoding, China; ^3^The National Clinical Research Center for Mental Disorders & Beijing Key Laboratory of Mental Disorders, Beijing Anding Hospital & the Advanced Innovation Center for Human Brain Protection, Capital Medical University, Beijing, China; ^4^Unit of Psychiatry, Institute of Translational Medicine, Faculty of Health Sciences, University of Macau, Macao, China; ^5^Division of Psychiatry, School of Medicine, University of Western Australia, Perth, WA, Australia; ^6^University of Notre Dame, Fremantle, WA, Australia; ^7^Department of Psychology, University of Macau, Macao, China; ^8^Center for Cognition and Brain Sciences, University of Macau, Macao, China; ^9^Institute of Advanced Studies in Humanities and Social Sciences, University of Macau, Macao, China

**Keywords:** mental health, epidemiology, correlates, China, agriculture

## Abstract

**Objective:** Poor mental health is associated with impaired social functioning, lower quality of life, and increased risk of suicide and mortality. This study examined the prevalence of poor general mental health among older adults (aged 65 years and above) and its sociodemographic correlates in Hebei province, which is a predominantly agricultural area of China.

**Methods:** This epidemiological survey was conducted from April to August 2016. General mental health status was assessed using the 12-item General Health Questionnaire (GHQ-12).

**Results:** A total of 3,911 participants were included. The prevalence of poor mental health (defined as GHQ-12 total score ≥ 4) was 9.31% [95% confidence interval (CI): 8.4–10.2%]. Multivariable logistic regression analyses found that female gender [*P* < 0.001, odds ratio (OR) = 1.63, 95% CI: 1.29–2.07], lower education level (*P* = 0.048, OR = 1.33, 95% CI: 1.00–1.75), lower annual household income (*P* = 0.005, OR = 1.72, 95% CI: 1.17–2.51), presence of major medical conditions (*P* < 0.001, OR = 2.95, 95% CI: 2.19–3.96) and family history of psychiatric disorders (*P* < 0.001, OR = 3.53, 95% CI: 2.02–6.17) were significantly associated with poor mental health.

**Conclusion:** The prevalence of poor mental health among older adults in a predominantly agricultural area was lower than findings from many other countries and areas in China. However, continued surveillance of mental health status among older adults in China is still needed.

## Introduction

The proportion of aging populations has been rapidly increasing in many nations, especially in developing countries (1, 2). For instance, the number of adults over age 65 is expected to increase globally from 703 million to 1.5 billion between 2019 and 2050 (from 9 to 16% of the total population) (1). In China, the percentage of individuals aged 65 years and above is expected to increase from 8.2% in 2010 to 23.3% by 2050 (3). In light of such trends, mental health among older adults has received growing attention in past decades due to its associations with daily activity and social functioning (3, 4). Compared with younger adults, older adults are more likely to experience negative life events such as living alone, decreased income after retirement, bereavement, poor social support and major medical conditions (5–9), all of which could lead to poor mental health.

Poor mental health is a widely used general health outcome that often reflects probable psychiatric morbidities or psychological distress (4, 10, 11). General mental health can be measured by structured interview protocols, such as the revised Clinical Interview Schedule (12) and Mini International Neuropsychiatric Interview (13) or self-report instruments including the General Health Questionnaire (GHQ) (14), the Affect Balance Scale (ABS) (15), the Symptom Checklist (SCL) (16) and the Patient Health Questionnaire (PHQ) (17). Among self-report instruments, the 12-item GHQ (GHQ-12) is one of the most widely used tools in epidemiological surveys (14, 18), and has been well validated as a measure of psychiatric symptoms related to depression and anxiety, social dysfunction and lack of confidence (19, 20). Negative health outcomes of poor mental health include cognitive dysfunction, impaired social functioning, lower quality of life, and even increased risk of suicide and mortality (21–24).

To better understand the impact of poor mental health on daily life and develop appropriate interventions to reduce negative outcomes for older adults, it is important to examine the prevalence of poor mental health and its correlates within this population. Previous studies have estimated the prevalence of poor mental health among the elderly to be 12.3% in nine European Union countries (6, 25), 8.3% in the United States (23), 28.4% in Japan (26), and 38.5% in Brazil (27). In China, corresponding figures have been 24.7% in Hong Kong (28), 23.8% in Jilin province (29) and 11.8% in Shanxi province (10). Discrepant findings between studies could be due partly to different measures of mental health status and sociocultural or economic factors.

Commonly identified correlates of poor mental health status among older adults include unhealthy lifestyles and behavior habits, chronic physical diseases, and poor living capabilities, disturbed family relationships and reduced social support, as well as demographic factors, particularly female gender and lower income, pre-retirement occupational status and/or education levels (5, 8, 10, 29–33).

The pattern of poor mental health and its correlates is closely associated with sociocultural and economic contexts (4, 29, 34). To date, however, little is known about the mental health status of older adults living in agricultural areas. This gap provided us with the impetus to investigate the prevalence of poor general mental health and its associated sociodemographic correlates among older adults in Hebei province, which is a predominantly agricultural area of China. Based on previous findings (10, 27, 29, 34), we hypothesized that the prevalence of poor mental health among older adults in Hebei province would differ from rates observed in urban areas of China due to differences in socioeconomic contexts. Examining this issue is expected to help healthcare professionals and policymakers develop appropriate intervention and prevention measures for poor mental health and develop routine screening procedures for older adults within agricultural regions.

## Methods

### Subjects and Sampling

This study was part of a large-scale cross-sectional study on mental health conducted in Hebei province from April to August 2016 (35). Hebei province is a predominantly agricultural area with a population of ~74.25 million people in 11 administrative regions (36). In 2014, the gross domestic product (GDP) of Hebei province was 2.942 trillion yuan (approximately USD $479 billion), and ranked sixth richest of 32 provinces in mainland China (37). With rapid economic expansion and significant advances in health care, the prevalence of older adults in Hebei province reached 14.96 million and represented 19.8% of the total population in 2018 (38). The study protocol was reviewed and approved by the Hebei Mental Health Centre IRB. Written informed consent was provided by all participants.

The sample size was calculated using the program OpenEpi according to the formula (39).

$$\begin{array}{l} n=\frac{DEFF\;*\;Np\left(1-p\right)}{\frac{d^{2}}{Z_{1-\alpha /2}^{2}}\;*\;\left(N-1\right)+p\;*\;\left(1-p\right)} \end{array}$$

In this formula, N was the total population in Hebei province. Given a design effect (DEFF) of 2.0, *Z*_1−α/2_ of 2.58 (level of significance 99% with the two-tailed test), *p* [the prevalence of any type of psychiatric disorder (18.51%) in an earlier survey in Hebei province (40)], and *d* (the precision of the estimate: 0.1p), the recommended sample size was at least 20,013. Assuming a response rate of 80%, the total sample initially solicited was 24,000.

Study inclusion criteria were (1) permanent residence in Hebei province, (2) age of 18 years or older, (3) ability to understand written Mandarin language and (4) willingness to complete the assessment. To obtain a representative sample of the general population, we used a multistage, stratified, cluster random sampling method to select participants from neighborhood communities of urban areas and villages of rural areas. This study included all 11 administrative regions of Hebei province. Following an earlier epidemiological survey in Hebei province (40), and taking into consideration the population ratio of urban to rural areas, 1–4 districts and 1–7 towns were randomly selected by a computer-generated random numbers table in each administrative region. Details of sampling methods have been elaborated elsewhere (35). Eventually, 24,000 eligible residents from 20 communities in urban areas and 58 villages in rural regions were randomly selected.

### Assessment Tools and Procedure

A data collection sheet designed for this study was used to record basic socio-demographic information and clinical characteristics as follows: age, gender, marital status, residential area, education level, employment status, annual household income, co-living status, religious beliefs, health insurance, presence of major medical conditions (e.g., hypertension, diabetes, cerebrovascular disease, cancer, and gastrointestinal diseases) and family history of psychiatric disorders.

General mental health status was assessed using the Chinese version of the 12-item General Health Questionnaire (GHQ-12) (41). The GHQ-12 is a self-administered scale to measure general mental health status (42), and has been widely used in many countries and satisfactory psychometric properties (41, 43). GHQ-12 items are scored from “not at all = 0” and “same as usual = 0” to “rather more than usual = 1” and “much more than usual = 1.” GHQ-12 total scores were calculated by summing all item scores, with higher total scores indicating poorer mental health status. Validity of the GHQ-12 Chinese Version has been tested in Taiwan and mainland China (41, 43). In China, GHQ-12 total scores of “≥ 4” have been used as the cut-off value for poor mental health, with a sensitivity of 76.85% and a specificity of 73.82% (43).

### Statistical Analyses

A database was established with the Epi data program (Version 3.1, Odense, Denmark). Data analyses were performed with SPSS, Version 24.0 (IBM SPSS, IBM Crop., Armonk, NY, USA). Socio-demographic data and clinical characteristics were compared between good and poor mental health groups using Chi-square tests, independent samples *t*-tests and Mann-Whitney U tests, as appropriate. A one-sample Kolmogorov-Smirnov test was used to check distribution normality of continuous variables. A multiple logistic regression analysis based on the “Enter” method was performed to examine independent correlates on poor mental health; mental health status (poor vs. good) was the dependent variable and those having significant group differences were found in preceding univariate analyses as independent variables. Statistical significance was set at 0.05 based on two-tailed tests.

## Results

From 23,675 persons (aged ≥18 years) invited to participate in the study, 20,884 completed the assessment, resulting in a participation rate of 88.2%. Of these, 3,911 persons aged 65 years and above (males: 1,892; females: 2,019) met all selection criteria and were included in this study. From this subset, the prevalence of poor mental health was estimated at 9.31% [95% confidence interval (CI): 8.4–10.2%].

Table 1 presents socio-demographic characteristics of participants based on mental health status. Good vs. poor mental health groups differed on gender, marital status, residential area, education level, employment status, annual household income, co-living status, presence of major medical condition and family history of psychiatric disorders (all *P*-values < 0.05).

**Table 1:** Socio-demographic characteristics of the study population (*N* = 3,911) by mental health status.

**Variables**	**Total sample (*****N*** **=** **3,911)**		**Those with good mental health (*****N*** **=** **3,547)**		**Those with poor mental health (*****N*** **=** **364)**		**Statistics**		
	***n***	**%**	***n***	**%**	***n***	**%**	***X^**2**^***	**df**	***P***
Age (years)							1.77	2	0.412
65–74 (mean age: 68.64 ± 2.74)	2,754	70.4	2,488	70.1	266	73.1			
75–84 (mean age: 78.73 ± 2.80)	989	25.3	903	25.5	86	23.6			
≥ 85 (mean age: 87.42 ± 2.56)	168	4.3	156	4.4	12	3.3			
Male	1,892	48.4	1,765	49.8	127	34.9	29.23	1	**<0.001**
Married/cohabiting	3,036	77.6	2,771	78.1	265	72.8	5.38	1	**0.020**
Urban area	826	21.1	778	21.9	48	13.2	15.16	1	**<0.001**
Education level							19.21	1	**<0.001**
Primary school or below^a^	2,640	67.5	2,357	66.5	283	77.7			
Secondary school or higher	1,271	32.5	1,190	33.5	81	22.3			
Unemployed	1,379	35.3	1,268	35.7	111	30.5	3.99	1	**0.046**
Low income^b^	3,016	77.1	2,695	76.0	321	88.2	27.88	1	**<0.001**
Living alone	642	16.4	568	16.0	74	20.3	4.48	1	**0.034**
Religious beliefs	268	6.9	241	6.8	27	7.4	0.20	1	0.654
Health insurance	3,822	97.7	3,467	97.7	355	97.5	0.70	1	0.791
Major medical conditions^c^	2,572	65.8	2,264	63.8	308	84.6	63.35	1	**<0.001**
Family history of psychiatric disorders	70	1.8	51	1.4	19	5.2	26.86	1	**<0.001**
	Mean	SD	Mean	SD	Mean	SD	T	df	*P*
Age (years)	72.00	6.09	72.05	6.13	71.56	5.76	1.44	3,909	0.126

*Bolded values: <0.05; M, mean; SD, standard deviation*.

*Good mental health was defined as the 12-item General Health Questionnaire (GHQ-12) <4*.

a *Lower education = <7 years of education*.

b *Low income: annual household income < RMB 30,000 (~USD 4,242)*.

c *Major medical conditions included hypertension, diabetes, cerebrovascular disease, cancer, and gastrointestinal diseases*.

Table 2 shows independent correlates of poor mental health status. Female gender [*P* < 0.001, odds ratio (OR) = 1.63, 95% CI: 1.29–2.07], lower education level (*P* = 0.048, OR = 1.33, 95% CI: 1.00–1.75), lower annual household income (*P* = 0.005, OR = 1.72, 95% CI: 1.17–2.51), presence of major medical conditions (*P* < 0.001, OR = 2.95, 95% CI: 2.19–3.96) and family history of psychiatric disorders (*P* < 0.001, OR = 3.53, 95% CI: 2.02–6.17) were significant correlates of poor mental health, and accounted for 8% of the total variance of the prediction model for mental health status.

**Table 2:** Independent correlates of poor mental health based on a multiple logistic regression analysis.

**Variables**	**Multivariate regression analysis**		
	**OR**	**95% CI**	***P*-value**
Female gender	1.63	1.29–2.07	<**0.001**
Married/cohabiting	0.97	0.73–1.29	0.813
Rural region	1.37	0.93–1.99	0.108
Primary school education or below^a^	1.33	1.00–1.75	**0.048**
Unemployed	1.01	0.77–1.33	0.941
Low income^b^	1.72	1.17–2.51	**0.005**
Living alone	1.11	0.82–1.52	0.497
Major medical conditions^c^	2.95	2.19–3.96	<**0.001**
Family history of psychiatric disorders	3.53	2.02–6.17	<**0.001**

*Bolded values: <0.05; CI, confidential interval; OR, odds ratio*.

a Primary school education or below = <7 years of formal education.

b *Low income: annual household income < RMB 30,000 (~USD 4,242)*.

c *Major medical conditions included hypertension, diabetes, cerebrovascular disease, cancer, and gastrointestinal diseases*.

## Discussion

In this large-scale epidemiological study, we found that the prevalence of poor mental health status based on total GHQ-12 scores of ≥4 was 9.31% among older adults in Hebei province. Furthermore, poor mental health had significant associations with female gender, lower education and annual household income levels, presence of major medical conditions and family history of psychiatric disorders.

The prevalence of poor mental health among older adults found in this study (9.31%) was lower than rates reported in other countries that also used the GHQ-12, including Finland (15.3%) (44), Brazil (38.5%) (27) and Japan (male:28.4%, female:29.6%) (26). Our prevalence estimate was also lower than corresponding figures among older Chinese adults in Hong Kong (24.7%) (28) and Jilin province (23.8%) (29). The lower prevalence of poor mental health status in this study could be due to several factors. First, different operationalizations of “older adults,” such as ≥60 years (27–29) and ≥65 years (26, 44), have been used in different studies. In addition, GHQ-12 cutoff values for “poor mental health status” have varied from ≥3 (44) to ≥4 (26, 29) to ≥7 (28) between studies. Second, differences in study periods, sampling methods and statistical methods may have contributed to different findings. Third, Hebei province is primarily an agricultural area in China. Compared with older adults living in non-agricultural areas in China, older residents in agricultural areas such as Hebei, may have less daily living pressure, which could contribute to a reduced likelihood of poor mental health status (45, 46).

Moreover, with its rapid economic development and growing healthcare budget, mental health promotion campaigns and routine screening for mental health problems, such as insomnia, have been widely implemented in Hebei province over the past decades. Health promotion campaigns include the provision of basic mental health services in community clinics and regular screening for psychiatric symptoms and disorders, particularly depression, anxiety and suicide. In addition, social support and financial subsidies for older adults have generally improved in Hebei province (40). Furthermore, in most agricultural areas of China, there may be greater stigma and discrimination associated with psychiatric disorders (45, 47–49); therefore participants may have been less likely to disclose poor mental health status in assessments (8, 50–53).

Finally, Chinese traditions in agricultural areas may have a protective effect on mental health of older adults (54, 55). For instance, older adults usually live with family members in China; thus, they could have better social support than their counterparts in big cities, which could reduce the risk of poor mental health (56–58). Further, Chinese elderly are commonly well-respected and cared for by their families and children as traditional Chinese culture emphasizes filial piety and reverence for seniors (8).

Several socio-demographic characteristics were significant correlates of poor mental health status. Similar to previous findings (8, 9, 29), women were more likely to have poor mental health status in this study. In China, due to sociocultural and historical factors, older women typically have less education, lower socioeconomic status, fewer social resources and reduced leisure time (10), each of which may contribute to poor mental health status as we found in this study. Furthermore, some women are vulnerable to sex hormone disturbances. Obvious fluctuations or chronic imbalances in sex hormones may contribute to mental health problems (59). Hormonal changes can affect emotions and moods and are associated with fatigue and irritability, depression and anxiety (60).

Socioeconomic status, as measured by education, employment and/or income, is closely associated with mental health status. Some studies (29, 30, 32) have found that less educated persons and those with lower income often have a poorer self-reported health status in support of our findings. Older adults with higher levels of education and income are more likely to understand the importance of both physical and mental health, and have better access to healthcare services (28, 34), hence reducing their risk for poor mental health outcomes. Previous studies have also found that lower socioeconomic status is associated with malnutrition, undiagnosed physical diseases and more frequent adverse life events (11), all of which could increase risk of mental health problems.

Consistent with other studies (8, 29, 61), presence of major medical conditions and family history of psychiatric disorders were also significant correlates of increased risk for poor mental health status. In this study, major medical conditions included hypertension, diabetes, cerebrovascular disease, cancer, and gastrointestinal diseases, all of which have been linked with poor mental health status (62, 63). These medical conditions are frequently accompanied by somatic discomfort, chronic pain, restrictions in daily activities and heavy economic burdens, which can lower quality of life and increase risk for mental health problems (64). Given that many psychiatric disorders have a substantial genetic component, older adults who report a family history of psychiatric disorders are more likely to have poor mental health status.

Strengths of this study included its consideration of an understudied population (i.e., older adults living in a major agricultural region of China), a large sample size, multistage random sampling, and use of a standardized, widely-employed instrument to evaluate mental health status. Nonetheless, results should be interpreted with caution due to several methodological limitations. First, because the GHQ-12 is a tool designed to assess general mental health, diagnosis of specific psychiatric disorders could not be made. Second, causal relations between poor mental health status and respondent demographics and clinical characteristics could not be examined due to cross-sectional, non-experimental study design. Third, potentially important correlates of mental health, such as social support were not assessed in this study. Fourth, some older adults were excluded because they could not complete the assessment due to vision problems, hearing loss and/or poor cognitive functioning, all of which could lead to a potential selection bias toward healthier respondents. Finally, clinical information, such as the presence of major medical diagnoses was self-reported by participants and confirmed with their family members; therefore the possibility of recall biases cannot be discounted.

## Conclusion

In conclusion, the prevalence of poor mental health among older adults in Hebei province was lower than rates reported in other countries and regions of China. As such, factors contributing to the lower prevalence of poor mental health in agricultural areas should be explored in future longitudinal studies because such data may be helpful in guiding healthcare policy in other parts of China. In addition, due to the negative impact of poor mental health status on daily functioning and quality of life, continued surveillance of mental health status among older adults is needed in Hebei province and other understudied agricultural areas of China.

## Data Availability Statement

The Clinical Research Ethics Committee of Hebei Mental Health Hospital that approved the study prohibits the authors from making the research data set publicly available. Readers and all interested researchers may contact Dr. Ke-Qing Li (Email address: likel002@sina.com) for details. Dr. Ke-Qing Li could apply to the Clinical Research Ethics Committee of Hebei Mental Health Hospital for the release of the data.

## Ethics Statement

The studies involving human participants were reviewed and approved by Hebei Mental Health Centre IRB. The patients/participants provided their written informed consent to participate in this study.

## Author Contributions

Y-SZ, YJ, K-QL, and Y-TX: study design. YJ, W-WR, QZ, L-LZ, L-JC, J-FL, and LL: collection, analysis, and interpretation of data. Y-SZ, YJ, and Y-TX: drafting of the manuscript. GU and TJ: critical revision of the manuscript. All authors contributed to the article and approved the submitted version.

## Conflict of Interest

The authors declare that the research was conducted in the absence of any commercial or financial relationships that could be construed as a potential conflict of interest.
