# Corrigendum: Prevalence and Socio-Demographic Correlates of Poor Mental Health Among Older Adults in Agricultural Areas of China

**DOI:** 10.3389/fpsyt.2021.664145

**Published:** 2021-03-10

**Authors:** Yu Jin, Yun-Shu Zhang, Qinge Zhang, Wen-Wang Rao, Li-Li Zhang, Li-Jun Cui, Jian-Feng Li, Lin Li, Gabor S. Ungvari, Todd Jackson, Ke-Qing Li, Yu-Tao Xiang

**Affiliations:** ^1^College of Education for the Future, Beijing Normal University at Zhuhai, Zhuhai, China; ^2^Department of Sleep Medicine, Hebei Psychiatric Hospital, Baoding, China; ^3^The National Clinical Research Center for Mental Disorders & Beijing Key Laboratory of Mental Disorders, Beijing Anding Hospital & the Advanced Innovation Center for Human Brain Protection, Capital Medical University, Beijing, China; ^4^Unit of Psychiatry, Institute of Translational Medicine, Faculty of Health Sciences, University of Macau, Macao, China; ^5^Division of Psychiatry, School of Medicine, University of Western Australia, Perth, WA, Australia; ^6^University of Notre Dame, Fremantle, WA, Australia; ^7^Department of Psychology, University of Macau, Macao, China; ^8^Center for Cognition and Brain Sciences, University of Macau, Macao, China; ^9^Institute of Advanced Studies in Humanities and Social Sciences, University of Macau, Macao, China

**Keywords:** mental health, epidemiology, correlates, China, agriculture

In this published article, there was an error in the affiliation ^**^1^**^. Instead of “^**^Faculty of Science, Kunming University of Science and Technology, Kunming, China^**^”, it should be “^**^College of Education for the Future, Beijing Normal University at Zhuhai, Zhuhai, China^**^”.

The authors apologize for this error and state that this does not change the scientific conclusions of the article in any way. The original article has been updated.

